# Liposomal delivery of gene therapy for ovarian cancer: a systematic review

**DOI:** 10.1186/s12958-023-01125-2

**Published:** 2023-08-23

**Authors:** Jin Sung Son, Ryan Chow, Helena Kim, Toney Lieu, Maria Xiao, Sunny Kim, Kathy Matuszewska, Madison Pereira, David Le Nguyen, Jim Petrik

**Affiliations:** 1 Faculty of Health Sciences, University of McMaster, Hamilton, ON Canada; 2 Faculty of Medicine, University of Ottawa, Ottawa, ON Canada; 3 Department of Biomedical Sciences, University of Guelph, Guelph, ON Canada; 4 Department of Obstetrics and Gynecology, University of McMaster, Hamilton, ON Canada

**Keywords:** Ovarian cancer, Liposomes, Gene therapy, micro RNAs

## Abstract

**Objective:**

To systematically identify and narratively synthesize the evidence surrounding liposomal delivery of gene therapy and the outcome for ovarian cancer.

**Methods:**

An electronic database search of the Embase, MEDLINE and Web of Science from inception until July 7, 2023, was conducted to identify primary studies that investigated the effect of liposomal delivery of gene therapy on ovarian cancer outcomes. Retrieved studies were assessed against the eligibility criteria for inclusion.

**Results:**

The search yielded 564 studies, of which 75 met the inclusion criteria. Four major types of liposomes were identified: cationic, neutral, polymer-coated, and ligand-targeted liposomes. The liposome with the most evidence involved cationic liposomes which are characterized by their positively charged phospholipids (n = 37, 49.3%). Similarly, those with neutrally charged phospholipids, such as 1,2-dioleoyl-sn-glycero-3-phosphatidylcholine, were highly researched as well (n = 25, 33.3%). Eight areas of gene therapy research were identified, evaluating either target proteins/transcripts or molecular pathways: microRNAs, ephrin type-A receptor 2 (EphA2), interleukins, mitogen-activated protein kinase (MAPK), human-telomerase reverse transcriptase/E1A (hTERT/EA1), suicide gene, p53, and multidrug resistance mutation 1 (MDR1).

**Conclusion:**

Liposomal delivery of gene therapy for ovarian cancer shows promise in many in vivo studies. Emerging polymer-coated and ligand-targeted liposomes have been gaining interest as they have been shown to have more stability and specificity. We found that gene therapy involving microRNAs was the most frequently studied. Overall, liposomal genetic therapy has been shown to reduce tumor size and weight and improve survivability. More research involving the delivery and targets of gene therapy for ovarian cancer may be a promising avenue to improve patient outcomes.

**Supplementary Information:**

The online version contains supplementary material available at 10.1186/s12958-023-01125-2.

## Introduction

Ovarian cancer is the deadliest gynecologic cancer and the fifth leading cause of female cancer deaths with 19,710 projected new diagnoses and 13,270 projected deaths in the United States for 2023 [1]. In comparison with cervical and breast cancer that have a five-year survival rate of 66% and 90% respectively, ovarian cancer presents a relatively low five-year survival rate of 45.6% [2–4]. This poor prognosis can be attributed to the lack of subjective symptoms, inadequate screening tests, and inefficient therapeutic measures for ovarian cancer, as more than 75% of affected women are diagnosed at an advanced stage (III or IV) that presents a lower five-year survival rate of 17-28% [5]. The current standard of care, primary cytoreductive surgery and adjuvant platinum-based chemotherapy, is greatly hindered by the high recurrence rate of 80% and the accompanying drug resistance [6–8]. In turn, novel ovarian cancer therapeutic modalities to improve clinical outcomes and survival for patients need to be explored.

A potential new therapeutic avenue for ovarian cancer is gene therapy, a technique known to replace, inactivate, or induce genes in patients’ cells [9]. Within gene therapy, there are several different approaches and strategies to potentially treat cancer. For instance, emerging RNA interference (RNAi) technologies, such as small-interfering RNA (siRNA), are being developed to silence ovarian cancer oncogenes and mutated tumor suppressor genes by inactivating its complementary mRNA. Therapeutics like siRNA demonstrate high specificity, high efficiency, and low toxicity, but are prone to degradation and lack cell membrane permeability [10]. However, effective accumulation and cell membrane permeability can be achieved through efficient gene carriers. This study’s focus is on liposomes as they are known to effectively deliver cancer therapeutics to effector sites. Currently, liposomes are the most commonly investigated nanocarrier and the first therapeutic nanoparticle clinically approved for cancer treatment [11]. The widespread representation of liposomes in nanotherapeutics can be accredited to its unique characteristics and properties serving several advantages. As a nanocarrier prepared from naturally derived phospholipids that mimic the mammalian cell membrane, liposomes are non-toxic, biocompatible, biodegradable, and non-immunogenic in nature. Furthermore, as liposomes increase cellular uptake and efficacy of encapsulated therapeutics, drug clearance and degradation are reduced [12].

Several studies have indicated promising outcomes involving ovarian cancer when utilizing liposomes in combination with different types of gene therapies. Despite the need for further investigation on liposome-encapsulated gene therapy, there has been no synthesis of evidence on this topic. Therefore, the aim of this systematic review is to summarize the evidence surrounding liposomal gene therapy and its implications on ovarian cancer. Furthermore, this systematic review hopes to identify gaps in the literature and the most promising applications of gene therapy in this field.

## Methods

This systematic review was conducted according to the Preferred Reporting Items of Systematic Reviews and Meta-Analyses (PRISMA) guidelines [13]. The study protocol was registered in the PROSPERO international prospective register of systematic review (CRD42021233736).

### Search strategy

A comprehensive electronic database search of Embase, MEDLINE and Web of Science from inception until July 7, 2023, was conducted. A medical librarian aided in generating and validating a search strategy. Search terms include, “ovarian cancer”, “liposomes”, and “gene therapy”. The complete search strategy is available in the appendix.

### Study selection

Search results were uploaded into the Covidence software platform (Veritas Health Innovation Ltd.). Duplicate articles were automatically removed, and an independent screening process was used to identify studies for inclusion. Pilots were run for the initial stage of screening until review authors (H.K., T.L., M.X., and S.K.) reached a kappa agreement value of 0.8. Reviewers then independently screened titles and abstracts. Eligible articles proceeded to the next stage, full-text screening. Discrepancies during either stage of screening were resolved by discussion among the authorship team until a consensus was reached. The inclusion criteria involved: [1] studies must focus on ovarian cancer patients, animal models, or cell-lines; [2] studies must examine the interventions of liposomes and gene therapy; [3] studies must report on the impact of liposomal gene therapy on the outcomes of ovarian cancer progression. The exclusion criteria involved: (1) conference or abstract submissions; (2) reviews or systematic reviews; (3) non-English literature.

### Data extraction

Data was independently extracted by authors (H.K., T.L., M.X., and S.K.). Domains extracted included publication details such as: journal, author, year of publication, liposome studied, intervention studied (which gene therapy), sample characteristics (which model), sample size (n), and measured outcomes (i.e., mean difference, standard deviation, Pearson’s correlation/R, bivariate analysis, P values). Discrepancies were resolved by discussion among the authorship team until a consensus was reached.

## Results

### Study selection

**Fig. 1:**
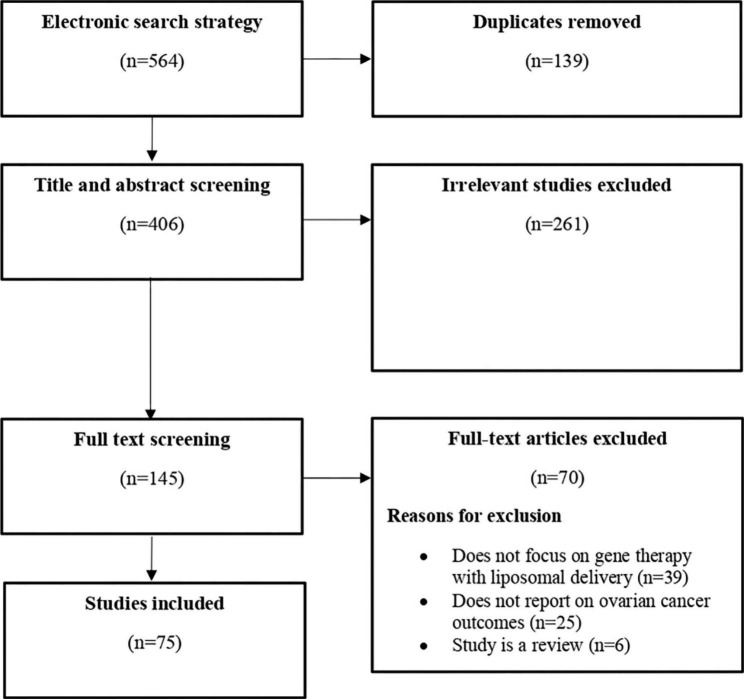
PRISMA flow diagram for study selection: PRISMA flow diagram for the literature on liposomal gene therapy and ovarian cancer from inception to July 2023

The electronic searches identified 564 publications, 139 (25%) of which were duplicates (Fig. 1). 406 articles proceeded to the title/abstract screening with 261 (49%) being deemed ineligible. 145 (26%) full-text articles were retrieved and subjected to another round of screening from which 70 (12%) studies were excluded. Specifically, 39 (7%) studies did not examine gene therapy, 25 (4.4%) studies did not report ovarian cancer outcomes, and six (1%) studies were reviews. Finally, 75 studies (13%) met the *a priori* inclusion criteria. The list of included studies is available in the appendix (Appendix S1).

### Study characteristics

**Fig. 2:**
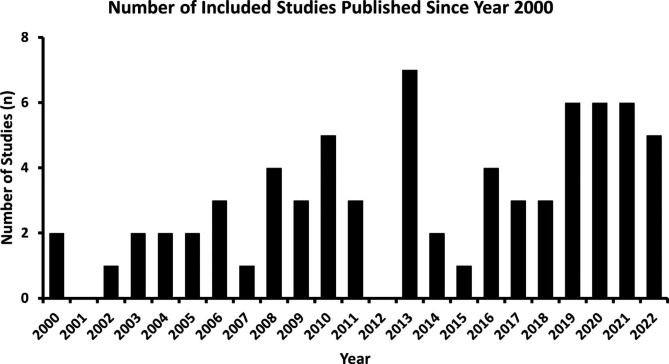
Number of included studies published per year since the year 2000: The years with the most included studies on liposomes and gene therapy for ovarian cancer in the last 20 years were 2013 (n = 7), 2019 (n = 6), 2020 (n = 6), and 2021 (n = 6)

Prior to the year 2000, the body of research concerning liposomal delivery of gene therapy and its impact on ovarian cancer was limited (n = 3). However, subsequent to this timeframe, there were noticeable trends in the publication years of the included studies encompassing this subject matter. Overall, there was a gradual upward trend of published studies, most notably in the recent years from 2015 to 2022 (Fig. 2). The greatest number of studies were published in 2013 (n = 7, 9%) and the least number of studies were published in 2001 and 2012 (n = 0).

**Fig. 3:**
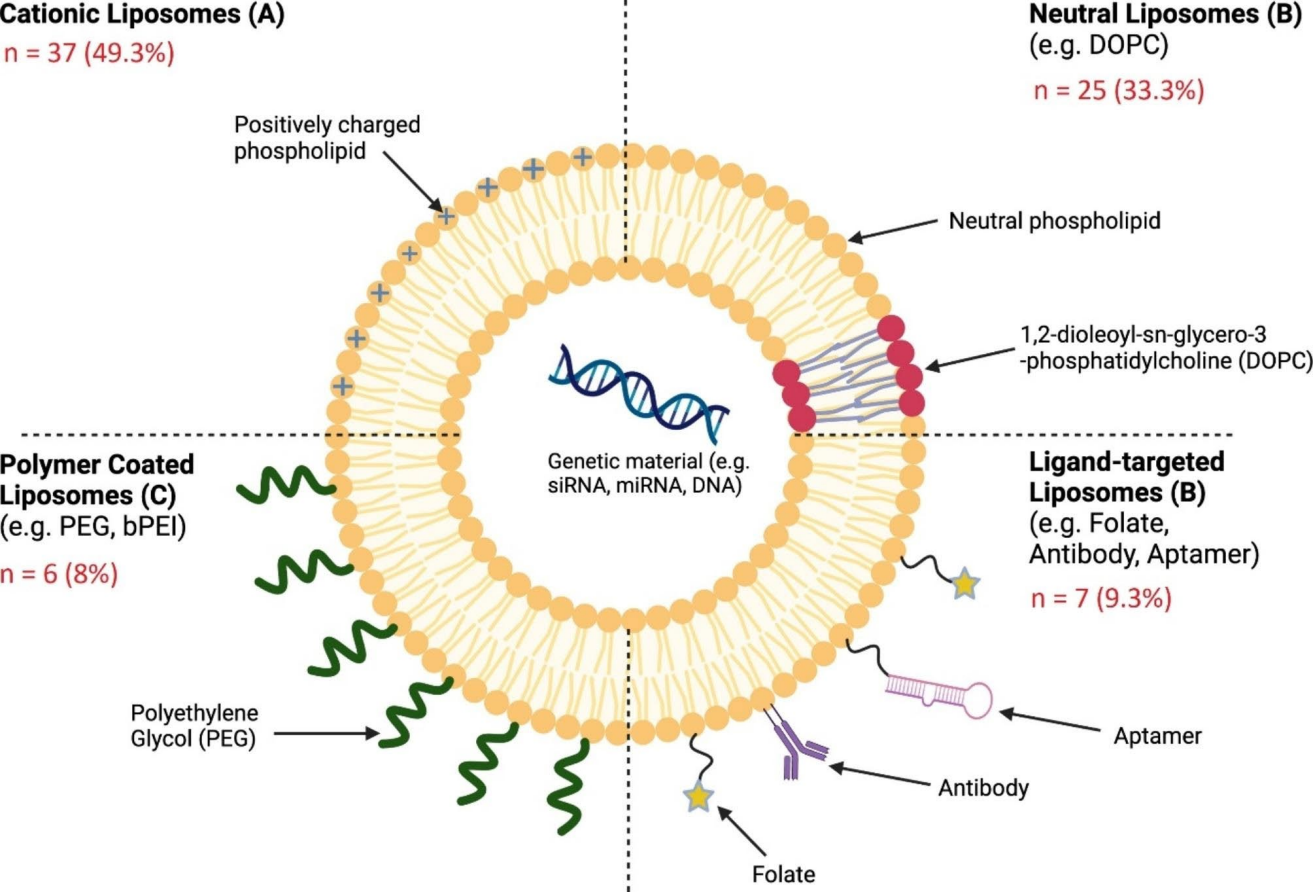
Distribution of studies that examined different liposomes: Distribution of included studies that examined the different types of liposomes used for gene therapy. The most extensively studied liposomes were the cationic liposomes (n = 37) and neutral liposomes (n = 25). DOPC, 1,2-dioleoyl-sn-glycero-3-phosphocholine; PEG, polyethylene glycol; bPEI, branched polyethyleneimine; siRNA, small interfering ribonucleic acid; micro-ribonucleic acid; DNA, deoxyribonucleic acid

Various types of liposomes were used to deliver therapeutic genes in the included studies. The four main categories include: cationic liposomes, neutral liposomes, polymer-coated liposomes, and ligand-targeted liposomes (Fig. 3). The most investigated delivery method was cationic liposomes which are characterized by their positively charged phospholipids (n = 37, 49.3%). Similarly, those with neutrally charged phospholipids, such as 1,2-dioleoyl-sn-glycero-3-phosphatidylcholine (DOPC), were highly prevalent as well (n = 25, 33.3%). There were also trends in the publication years for liposomal delivery with many of the recent studies examining polymer or antibody conjugated liposomes (n = 6, 8% and n = 7, 9.3% respectively).

**Fig. 4:**
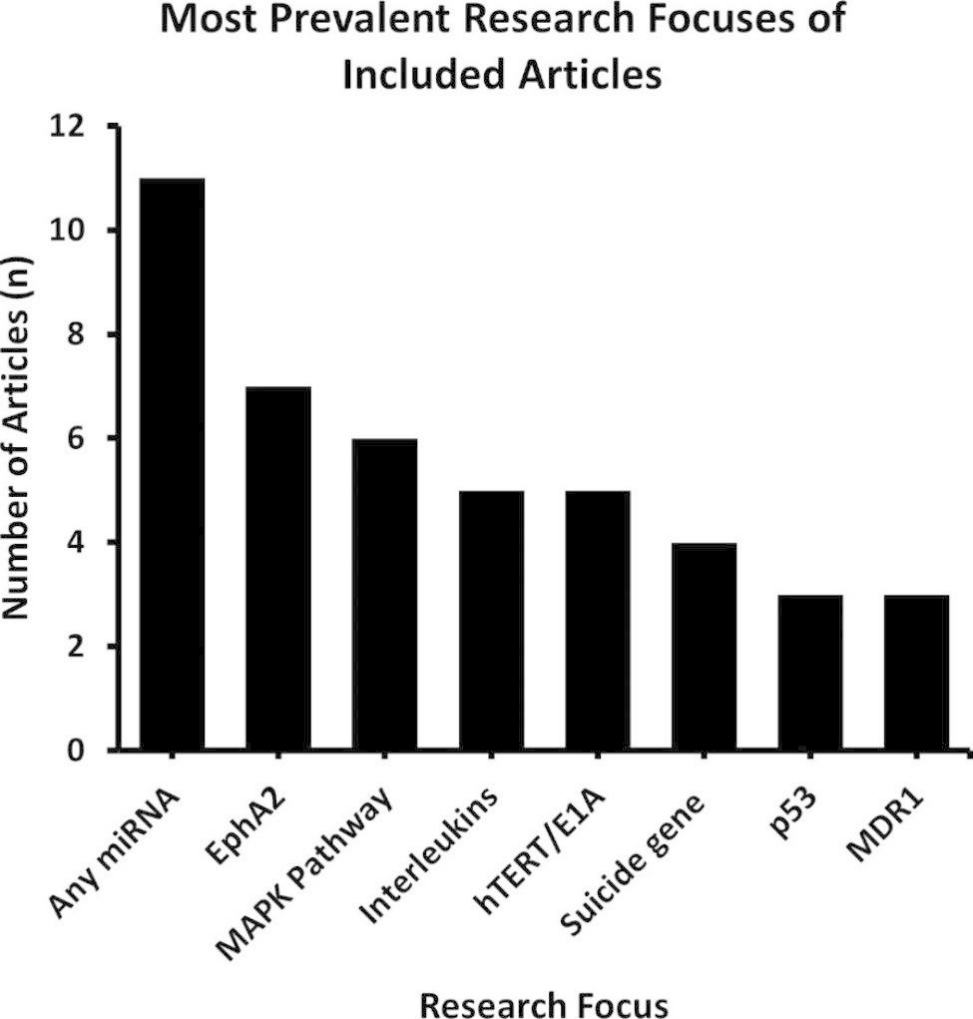
Number of included studies that examined genes or pathways and their associated functions in the treatment of ovarian cancer: The most extensively studied gene and its associated pathway was miRNA (n = 11), followed by various EphA2 (n = 7), different MAPK (n = 6), then interleukins (n = 5) and hTERT/E1A (n = 5). miRNA, micro-ribonucleic acid; EphA2, ephrin type-A receptor 2; MAPK, mitogen-activated protein kinase; hTERT/E1A, human-telomerase reverse transcriptase/E1A; MDR1, multidrug resistance mutation 1

There were common themes observed for gene therapy research throughout the range of included studies. The eight most highly researched areas include: microRNAs (miRNAs), EphA2, interleukins, MAPK, hTERT/EA1, suicide gene, p53, and MDR1 (Fig. 4). Of the most common interventions studied, microRNA was the most examined (n = 11, 15%). Studies that investigated EphA2 targets in the context of liposomal gene therapy were also highly prevalent (n = 7, 9%). Targets in immune pathways involving the MAPK pathway (n = 6, 8%) and interleukins and hTERT/E1A (n = 5, 7%) were examined in-depth as well. Individual study characteristics and breakdown of objectives and main findings are available in the appendix (Table S1).

## Discussion

Ovarian cancer is the leading cause of death in women diagnosed with gynecological cancer and lacks effective treatment options [14]. Currently, gene therapy is an emerging therapy that may assist in overcoming this barrier and it has shown promise in effectively treating ovarian cancer. Although a significant challenge arises in the delivery of the treatment, liposome carriers can be used to protect the gene from the body’s immune system and navigate the “genetic medication” to specific tumor sites [12]. A liposome is composed of a phospholipid bilayer with a hydrophilic and hydrophobic section that separates the encapsulated genes from the external environment where it is prone to degradation [15]. While all liposomes share the same bilayer skeleton, many liposomes express different structures and compositions. Accordingly, different types of liposomes possess unique properties, and these variations can be categorized into four major groups: neutral liposomes, cationic liposomes, pegylated liposomes, and ligand-targeted liposomes.

In our included studies, the most investigated liposomes were cationic and neutral liposomes. Cationic liposomes are made of positively charged phospholipids, namely dioleoyl-3-trimethylammonium propane (DOTAP) and dioleoyl-phosphatidylethanolamine (DOPE) [16]. It can undergo stabilizing electrostatic interactions with the negatively charged nucleic acids and cell membranes to improve the transportation of large DNA or RNA, while limiting liposome aggregation in blood [17, 18]. While there are several advantages to the electrostatic interactions of the cationic liposomes, they can also serve as a hindrance. The positively charged phospholipid can interact with the surface proteins of tumor cells and generate an electrostatically derived binding site barrier that limits the penetration of the gene to tumor sites [17]. This limitation is absent in neutral liposomes that consist of neutrally charged zwitterionic phospholipids such as DOPC [16]. Compared to cationic DOTAP liposomes and naked siRNA, one study found that DOPC liposomes demonstrated a 10-fold and 30-fold improvement in siRNA delivery, respectively [19]. It is proposed that the neutral charge helps to reduce protein binding and uptake efficiency by endothelial cells to increase the plasma circulation time of the gene carrier [19]. However, the lack of surface charge limits stability by increasing the aggregation of liposomes in blood and interactions with target cells reducing its efficacy in suppressing tumor growth [18]. In general, conventional liposomes (cationic and neutral) are quickly captured by the reticuloendothelial system (RES) and accumulate in the spleen and liver, where it is unable to be delivered to target cells [20].

Newer liposomes serve to overcome this limitation and have been increasing in the interest of recently published studies. One approach gaining attention involves the surface modification of liposomes with polymers. By grafting neutrally charged polyethylene glycol (PEG) polymers to the surface of the conventional liposome, PEGylated liposomes significantly suppress interactions with serum protein and phagocytic cells, primarily Kupffer cells in the liver, to increase serum stability and avoid RES uptake and renal clearance [21, 22]. One study investigated the impact of PEGylation on DC-Chol/DOPE cationic liposomes in siRNA delivery by conducting biodistribution studies on mice bearing SKOV3 tumors. The PEGylated group demonstrated a significant reduction in fluorescence intensities in the kidneys and liver, along with a significant accumulation at the tumor tissue in comparison to its non-PEGylated counterpart [23]. However, limitations of PEG include the stimulation of IgM production after repetitive doses and the reduced interaction between the tumor cells and liposomes, resulting in poor and inefficient cellular internalization [24, 25]. An alternative surface polymer to PEG is the branched polyethyleneimine (bPEI). In one study, bPEI modified liposomes demonstrated enhanced cellular association by up to 75 times compared to noncoated or PEGylated liposomes in A2780-ADR and SKOV-3TR ovarian cancer cell lines [26]. Another strategy to improve liposomal delivery involves ligand-targeted surface proteins. Attaching targeting ligands on the surface of the liposomes induce selective and active targeting to specific tumor sites and enhance tumor uptake [27]. A ligand widely used in liposomes is folate [28–30]. This compound directs the carrier to the folate receptor α (FRα), which is overexpressed in 90% of ovarian carcinomas [31, 32]. Upon binding, the plasma membrane of the cancer cell undergoes invagination, leading to the internationalization of the liposome and formation of endosomes where the therapy can exert its intended effects [33]. In FRα-positive SKOV3 cells, the transfection efficiency was significantly higher in folate-targeted liposomes compared to plain liposomes (p < 0.001) [30]. Other notable ligand candidates that are used in liposomes include EGF, aptamer and integrin alpha-v and beta-3 [34–36].

Utilizing these different liposomal delivery methods, several types of gene therapies, with different genetic targets, were evaluated regarding ovarian cancer. The most well-researched gene therapy categories included: microRNAs (miRNAs) (n = 11), ephrin type-A receptor 2 (EphA2) (n = 7), mitogen-activated protein kinase (MAPK) pathway (n = 6), interleukins (n = 5), hTERT/E1A (n = 5), suicide gene (n = 4), p53 (n = 3), and MDR1 (n = 3).

miRNAs, small noncoding RNAs, function as post-transcriptional regulators of messenger RNA molecules that encodes protein. In the context of gene therapy, the expression and activity of miRNA can be regulated by inhibitors, such as antisense-oligonucleotides, or replaced by synthetic miRNAs and miRNA mimics [37]. Lately, miRNA therapy via liposomes is becoming increasingly more studied in the context of ovarian cancer. There have been eleven studies evaluating the therapeutic potential of different miRNAs [29, 34, 35, 38–45]. In one study, transfecting miR-124 or miR-152 mimics into SKOV3ip1 cells xenograft in a nude mice model, via cationic liposome, significantly decreased tumor volumes compared to control mice (7.88 ± 2.84 mm^3^ vs. 43.57 ± 20.64 mm^3^ and 8.64 ± 3.52 mm^3^ vs. 45.74 ± 22.31 mm^3^ respectively; p < 0.01) [38]. Promising outcomes were also observed when introducing miR-192 mimics in female athymic nude mice bearing SKOV3ip1 cells. The administration of miR-192 with neutral DOPC liposomes significantly decreased in tumor burden by 70% (p < 0.05) compared to control [40]. In another study, miRNA-106a mimics were used to determine its correlation with CDDP resistance in OVCAR3/CIS cells. The cationic liposome loaded miRNA-106a mimic was significantly associated with the ovarian cancer cell survival (p < 0.05), while the introduction of the miRNA-106a inhibitors lowered the survival rate of the tumor cell lines (p < 0.05) [39]. More recently, studies have been conjugating miRNAs with ligand targeted liposomes. In cisplatin-resistant ovarian cancer cells, miR-18a-oligonucleotide miRNA mimics loaded via folate targeted liposomes significantly reduced tumor weight and the number of nodules compared to control (p < 0.05) [29]. Another study conjugated integrin alpha-v and beta-3 targeted hybrid liposomes with miR497 and triptolide in BALB/c-nu mice bearing SKOV3-CDDP tumors and found significant suppression of tumor growth by 87% compared to the combination of naked miR497 and triptolide (p < 0.001) [34].

Eph receptors and ephrin (the Eph receptor-interacting ligand) join in a signalling network that has many functions [46]. The tyrosine kinase EphA2, belongs to the family of Eph receptors and is highly expressed in tumor cells. EphA2 is found at relatively lower levels in normal adult tissue, and this may indicate that it has potential for application in the treatment of cancer [46]. Eph receptors are single transmembrane potentials with intra- and extra (N-terminal) domains with ligand-binding and enzymatic activities. There is an accumulating body of evidence that EphA2 is abundantly expressed in ovarian cancer and is involved as an active participant in tumorigenesis [47]. One study found that EphA2 targeting siRNA-DOPC, significantly reduced tumor growth compared to control siRNA in SKOV3 mice (0.35 vs. 0.70 g; p = 0.020) [19]. Another study found that treatment with S1MP-EphA2- siRNA-DOPC in mice with SKOV3ip1 tumor cells significantly reduced tumor weight by 54.2% and 65.3% compared with non-silencing control siRNA-DOPC and S1MP- non-silencing control-siRNA-DOPC, respectively (p < 0.05; ANOVA F = 4.92) [48]. Three other studies demonstrated the same positive effect involving EphA2 siRNA gene therapy and this avenue of research should be further explored [42, 49, 50].

Mitogen-activated protein kinase (MAPK) pathways involve the interaction between a group of serine/threonine kinases that regulate several cellular functions, including cell proliferation, growth, differentiation, and apoptosis [51]. These important cellular processes are hyperactivated in ovarian cancer cells by mutations and abnormalities in the MAPK pathway and, consequently, play a vital role in its progression and development [52]. In our included studies, four studies examined different targets in the signalling cascade to suppress the development of ovarian cancer. The MAPK pathway begins with a signalling molecule that binds to a surface receptor such as the epithelial growth factor receptor (EFGR). Silencing this receptor with a T7 autogene-based hybrid mRNA/DNA system shEGFR via DOPC lipoplex in mice models demonstrated a 67% reduction in tumor weight compared to control (p < 0.001) [53]. The MAPK pathway can also be upregulated through another receptor, Prostaglandin E2 receptor EP3 (PTGER3). Mice treated with DOPC-PTGER3-siRNA showed a 40% reduction in cell proliferation compared to control (p < 0.001) [54]. Subsequently in the cascade, the growth factor receptor activates growth factor receptor-bound protein 2 (Grb2) that then activates the Ras protein, resulting in a series of phosphorylation events by the kinases. Inhibition of the Grb2 expression in mice via liposomal antisense oligodeoxynucleotide DOPC liposomes has shown decreased tumor growth (0.29 g ± 0.14 g, p < 0.05) and a greater decrease in tumor weight when treated in combination with Paclitaxel (0.82 g ± 0.25 g, p < 0.05) [55]. Further downstream of the signalling cascade leads to a transcription factor c-myc that plays a vital role in cell proliferation and regulation. Ovarian cancer cell lines treated with antisense phosphonothioate oligodeoxynucleotides targeting c-myc and c-erbb2 via cationic liposome reduced target gene expression and cell growth by 61.9 ± 9.3% and 64.5 ± 11.2%, respectively (p < 0.01) [56].

Interleukins and cytokines serve as a means of communication between innate and adaptive immune cells and the environment. Interleukins create an environment favouring cancer growth, whilst also being essential for an effective tumor-directed immune response [57]. Three interleukins have been studied a little more in-depth, namely IL-7, IL-8 and IL-12 in the context of liposomal delivery and ovarian cancer [58–61]. IL-7 gene was transfected into SKOV3 cells by nanoliposome, in severe combined immunodeficient (SCID) mice. They found that TGFbeta1 secretion was downregulated, ICAM-1 expression was upregulated and sensitivity to lymphokine-activated killer cells was enhanced (via LDH release assay) [58]. A study examining IL-8 siRNA-DOPC showed that SKOV3ip1 mice treated with IL-8 siRNA-DOPC had a 41% reduced tumor weight compared to controls (p < 0.006) [59]. Similar results were found in another study that showed IL-8 siRNA-DOPC reduced the mean tumor weight by 32% (95% CI = 14–50%; p = 0.03) and 52% (95% CI = 27–78%; p = 0.03) in the HeyA8 and SKOV3ip1 mice respectively [60]. One study examined co-transfection of IL-12 and (salmosin) Sal genes via anti-EGFR immunolipoplexes inhibited tumor growth and pulmonary metastasis (p < 0.001). Moreover, treatment with the anti-EGFR immunolipoplexes containing IL-12/ILSal, and doxorubicin significantly reduced tumor growth (p < 0.001) [61]. One chemokine was examined, CXCR4 siRNA liposome complexes, and this therapy showed a 39.2% reduction (0.398 ± 0.062 g; p < 0.01) in tumor weight compared to the 8.9% reduction (0.655 ± 0.034 g; p > 0.01) in the empty liposome control group [62].

Human telomerase reverse transcriptase (hTERT) is a catalytic subunit of telomerase, which, when combined with the telomerase RNA component (TERC), creates the most important unit of the telomerase complex [63]. hTERT catalyzes the addition of nucleotides in a TTAGGG sequence to the ends of a chromosome’s telomeres [64]. This prevents the degradation of the ends during DNA replication and has many associations with cancer [65]. One study examined the effect of hTERT shRNA in a SKOV3 cell model and found that it significantly increased the rate of tumor cell death compared to control (18.66 ± 1.33 vs. 2.92 ± 0.33; p < 0.05) [66]. However, most studies regarding hTERT utilize it as a promotor for E1A gene therapy. E1A is known to downregulate HER-2/neu overexpression commonly found in cancers and reverse the metastatic phenotype [67]. In one study, the cationic liposome delivery of hTERT-VISA-E1A into female athymic female BALB/c nu/nu mice with SKOV3ip1 tumor cells resulted in significant inhibition in tumor growth compared to the control vector (p < 0.05) [68]. Two studies found that the delivery of E1A via liposomal nanoparticles significantly reduces tumor growth in athymic female nu/nu mice models [69, 70].

In suicide gene therapy, or Gene-Directed Enzyme/Prodrug Therapy, suicide genes are introduced into specific cancer cells. These genes then encode enzymes that convert nontoxic prodrugs to cytotoxic metabolites, resulting in the death of the host cells [71]. Herpes Simplex Virus thymidine kinase (HSVtk) is a well documented suicide gene in treating ovarian cancer. HSVtk is a viral enzyme associated with the pyrimidine metabolic pathway and phosphorylates a series of nucleoside analogue such as the prodrug acyclovir (ACV) [72]. When female CD-1 nu/nu mice with KF-rb transplanted tumors (cisplatin-resistant tumor cells) were treated with novel cationic GTE321 or GTE319 liposome encapsulated HSVtk genes with 5 days cultivation of acyclovir, the growth of tumors were significantly reduced on day 32 (p < 0.05) [73]. The combination of HSVtk with a different prodrug, ganciclovir (GCV), yielded similar outcomes. The cationic liposome delivery of HSVtk gene and GCV in HRA or mEIIL cells xenograft CD-1 nu/nu mouse model significantly increased the mean survival time compared to control (76.9 days vs. 62.1 days respectively; p < 0.05) [74]. To improve the specificity for tumor cells, one study incorporated the human metallothionein IIa (hMTIIa) promoter in the HSVtk gene. The administration of liposomal encapsulated hMTIIa HSVtk gene to A2780 and A2780-E cisplatin-resistant cancer cells resulted in a 56-fold increase of TK mRNA and a significant sensitization to ganciclovir [75]. An alternative methodology employed in suicide gene therapy is cytosine deaminase/5-fluorocytosine (CD/5-FC) approach. In this strategy, the CD gene is engineered to produce a suicide enzyme that catalyzes the conversion of the prodrug 5-FC into the chemotherapeutic agent 5-fluorouracil [76]. The administration of cationic liposome encapsulated Survivin-VISA-hEndoyCD with 5-FC treatment in female athymic nude mice bearing SKOV3ip1-luc tumor cells significantly reduced the tumor weight compared to control (p < 0.01) [77].

TP*53* is a gene that encodes for the protein p53, which regulates the cell cycle as a tumor suppressor. It is important in multicellular organisms as it plays a vital role in suppressing the development of many cancers. It has been described as the “guardian of the genome,” due to its role in preventing genomic mutation [78]. *p53* is a transcriptional activator and regulates the expression of *MDM2, (*which in turn, regulates *p53*), and other genes associated with growth arrests such as *p21, Gadd45* and *14-3-3sigma*. It also plays a role in DNA repair by regulating *p53R2*, and apoptosis, *Bax, Apaf-1, PUMA and NoxA* [79]. In our included studies, three studies examined p53 directly [36, 41, 80]. Mice transduced with p53-plasmid-DNA with cationic liposomes showed a > 60% reduction in tumor volume compared to control [80]. Activating a p53 family member, TAp63, via miR-130b in DOPC liposomes has also been shown to decrease tumor burden in mice (p < 0.01) [41]. *p53* gene therapy has also been tested invitro, in SKOV3 cells, by delivery via anti-EGF antibody bound cationic polymeric liposomes and greatly enhanced cytotoxicity (p < 0.05) [36]. Survivin, another gene regulated by p53, has also been examined in two studies with cationic liposomes [77, 81]. The inhibition of survivin greatly reduced tumor weight in mice (p < 0.01) and significantly increased the inhibition rate of cellular proliferation compared to the negative control (7.0 ± 0.9 vs. 0.4 ± 1.8 respectively; p < 0.05) in SKOV3 cells [77, 81].

A few studies looked at the use of liposomal gene therapy in the context of drug-resistant and multi-drug-resistant cancers [26, 82, 83]. One study found that the use of modified liposomes with siRNA targeting MDR1, combined with paclitaxel, reduced tumor volumes by 40% (p < 0.0001) in female athymic nude mice bearing A2780ADR drug-resistant tumor cells [26]. Another study examined the use of antisense nucleotides targeting MDR1 and BCL2, combined with doxorubicin, delivered via a novel liposomal system. They found that the gene therapy led to significant overexpression of apoptotic protease activation factor-1, caspase 3 and caspase 9 in A2780ADR drug-resistant tumor cells [82]. The last study on this topic utilized a novel polycation coated liposome to carry siMDR1 to OVCAR8 and OVCAR8/ADR cell lines [83]. Significant tumor inhibition was detected and the IC50 of doxorubicin was 15 times lower than control [83].

Although the gene therapy approaches involving liposomal delivery have demonstrated encouraging results, it is equally important to recognize the accompanying challenges and limitations that warrant consideration. For example, miRNA is susceptible to off-target effects due to its expansive cellular effect, potentially giving rise to inefficiencies and unwanted side effects [84, 85]. Further investigation on active targeting and ligand targeted liposomes with miRNA may be warranted to enhance specificity and mitigate off-target effects. EphA2 possesses complex biological properties, and the precise mechanism of its forward and reverse signaling pathway is not fully elucidated [86]. The down regulation of EphA2 via siRNA liposomes can lead to compensatory stimulation of oncogenic signaling pathways and other Eph receptors, which can impact treatment effectiveness [46]. In the MAPK pathway, the use of EGFR inhibitors to ovarian cancer has seen limited success in clinical trials, possibly attributed to the scarcity of EGFR mutations in ovarian cancer which the inhibitor targets [87–89]. In suicide gene therapy, cancer-specific promoter, including hTERT, often exhibit limited transcriptional activity, resulting in inadequate activation of suicide genes to trigger the prodrugs [90]. Given the distinct strengths and challenges in the gene therapy approaches, alongside their biological complexities, there is no definitive gold standard gene therapy candidate. Moving forward, it is imperative that further research and accumulating evidence be pursued to identify a superior approach in the field of gene therapy and liposomal delivery.

## Conclusion

Liposomal delivery of gene therapy for ovarian cancer shows promise in many in vitro and in vivo studies. We determined that cationic liposomes were the most frequently investigated. Yet, emerging polymer-coated and ligand-targeted liposomes have been recently increasing in interest as it has been shown to further improve its stability and selective targeting to tumor sites. We found that gene-therapy involving miRNA was the most frequently studied intervention in the last 22 years. Other candidate targets that have been examined involve, p53, MAPK, hTERT/E1a, and EphA2 targets. Overall, liposomal genetic therapy has been shown to reduce tumor size, weight, and improve survivability. More research involving the delivery and targets of gene therapy for ovarian cancer may be a promising avenue to improve patient outcomes.

### Electronic supplementary material

Below is the link to the electronic supplementary material.

Additional File 1: Table S1. Characteristics of included studies.

## Data Availability

All of the data are contained within and available in this manuscript. Acknowledgements.
