# Supplementary material for: Liposomal delivery of gene therapy for ovarian cancer: a systematic review
Source: Reprod Biol Endocrinol. 2023 Aug 23;21:75. doi: 10.1186/s12958-023-01125-2 (PMC10464441; doi:10.1186/s12958-023-01125-2)
Supplement: Supplementary file 1 — Additional File 1: Table S1. Characteristics of included studies. [file 12958_2023_1125_MOESM1_ESM.docx]

| Title | Author (Year) | Journal | Liposome | Intervention Studied | Sample Characteristics (model) | Sample Size (n) | Measured Outcomes |
| --- | --- | --- | --- | --- | --- | --- | --- |
| Inhibition of human ovarian carcinoma cell proliferation by liposome-plasmid DNA complex | Hofland (1995) | Biochemical and Biophysical Research | Cationic liposome | liposome-plasmid DNA complex; using 3β[N-(N'N'-dimethylaminoethane)-carbamoyl] cholesterol (DC-chol) liposomes | human ovarian carcinoma 2008 cells xenograft to SCID mice | 5 mice/group | Mice treated with DNA-liposome comlex survived significantly longer 66.8 +/- 5 days (p < 0.001) compared to control mice (36.0 +/- 7.2 days; p < 0.01) |
| Liposome-mediated in vivo E1A gene transfer suppressed dissemination of ovarian cancer cells that overexpress HER-2/neu | Yu (1995) | Oncogene | Cationic liposome | E1A gene with cationic liposome | Female nu/nu mice bearing SKOV-3 cells that overexpressed HER-2/neu | Not reported | Liposome-mediated E1A gene transfer significantly inhibited growth and dissemination of ovarian cancer cells that overexpress HER-2/neu in the treated mice |
| Safety study and characterization of E1A–liposome complex gene-delivery protocol in an ovarian cancer model | Xing (1998) | Gene Therapy | Cationic liposome | mini-E1A with cationic liposomes | Athymic female homozygous nu/nu mice (4-6 weeks old) bearing SKOV-3.ip1 ovarian cell tumors | 5 mice/group | A DNA–liposome complex with a lower than standard lipid dose can transfer the gene with the same efficacy: The mice treated with the E1A–liposome complex at a 13:1 ratio survived significantly longer (E1A-13 versus PBS, P < 0.01). The treatment with E1A–liposome complex at a 1:1 ratio also prolonged mouse survival significantly (E1A-1 versus PBS, P < 0.01). |
| Nitric oxide-mediated tumor cell killing of cisplatin-based interferon-g gene therapy in murine ovarian carcinoma | Son (2000) | Cancer Gene Therapy Nature | Cationic liposome | IFN-g gene with cationic liposomes and cisplatin | C57BL6 WT or C57BL6 iNOSKO mice bearing MOT ovarian cell tumors | 12 mice/group | iNOSKO mice treated with both cisplatin and liposomal IFN-g gene did not produce a significant level of NO (12.1 6 4.5 mM), although WT mice receiving both cisplatin and liposomal IFN-g gene produced significant amounts of NO (113.7 6 17.9 mM). |
| Transactivation of the metallothionein promoter in cisplatin-resistant cancer cells: A specific gene therapy strategy | Vandier (2000) | Journal of the National Cancer Institute | Cationic liposome | pMT-TK plasmid with cationic liposomes | A2780(1A9) and A2780- E(80) (cisplatin resistant) cell lines | Not reported | No cytotoxicity was noted when cells transfected with pTK-b-gal were treated with ganciclovir at concentrations up to 50 mg/mL. Transfection of resistant cells with pMTTK or pCD3-TK resulted in marked sensitization to ganciclovir, with an IC50 of 20 mg/mL and 9 mg/mL, respectively. |
| Combination antigene therapy targeting c-myc and c-erbB2 in the ovarian cancer COC1 cell line | Fei (2002) | Gynecologic Oncology | Cationic liposome | liposmal antisense phosphorothioate oligodeoxynucleotides targeting c-erbB2 and c-myc (LF–c-erbB2/c-myc AS-ODNs) | COC1 cell line | 1x10^5 cells/well | Single antigene therapy reduced target gene expression and inhibited COC1 cell growth by 61.9 +/- 9.3 and 64.5 +/- 11.2% respectively; combination antigene therapy inhibited COC1 cell proliferation by 82.6 +/- 12.1% (p < 0.01). |
| Enhanced p53 gene transfer to human ovarian cancer cells using the cationic nonviral vector, DDC | Kim (2003) | Gynecologic Oncology | DDC cationic liposome | pp53-EGFP (p53 plasmid DNA) with DDC liposome | Female athymic nude mice (BALB/C) bearing OVCAR-3 ovarian tumor cells | 8 mice/group | Mice transduced with DDC/pp53-EGFP complexes showed a >60% reduction in tumor volume compared to control |
| Simultaneous modulation of multidrug resistance and antiapoptotic cellular defense by MDR1 and BCL-2 targeted antisense oligonucleotides enhances the anticancer efficacy of doxorubicin | Pakunlu (2003) | Pharmaceutical Research | DPPC liposome | DR1 and BCL-2 mRNA antisense oligonucleotide with DPPC liposomes and doxorubicin | A2780/AD | Not reported | Antisense oligonucleotides targeting BCL-2 and MDR1 mRNA with DOX led to significant overexpression of apoptotic protease activation factor-1 (APAF-1), caspases 3, and caspase 9 |
| A novel cancer therapy: combined liposomal hypoxia inducible factor 1 alpha antisense oligonucleotides and an anticancer drug | Wang (2004) | Biochemical Pharmacology | DOPC (1,2- dioleoyl-sn-glycero-3-phosphatidylcholine) liposome | HIF1A mRNA antisense oligonucleotides with DOPC liposomes and doxorubicin | A2780 and A2780/AD (multidrugresistant) cell lines | Not reported | Liposomal ASO targeted to HIF1A mRNA decreased the overexpression of the HIF1A gene and protein induced by hypoxia and DOX: concentrations of DOX corresponded to the IC50 doses for A2780 sensitive and A2780 multidrug-resistant cells equal to 0.2 and 3 mM, respectively. *P < 0.05 |
| Effect of interleukin-7 gene transfection into ovarian carcinoma cell line SKOV3 in vitro and in vivo | Xie (2004) | Gynecologic Oncology | Unreported | IL-7 gene with nanoliposome | Female SCID mice (4 and 6 weeks old )bearing SKOV3-IL-7, SKOV3-Neo and SKOV3 ovarian cell tumors | 3 mice/group | IL-7 level in peripheral blood of SCID mice inoculated with SKOV3-IL-7 was significantly higher than that in two parental untransfected control (10.810 ± 548 vs. 5.707 ± 2.217, P = 0.016) |
| Increased efficiency of cisplatin-resistant cell lines to DNA-mediated gene transfer with cationic liposome | Sato (2005) | Journal of Obstetrics and Gynaecology Research | Cationic liposome | Herpes simplex thymidine kinase (HSV-tk) gene with cationic liposomes and cultivation with aciclovir | KF and KFrb ovarian cancer cells xenograft to 5 weeks old female CD-1 nu/nu mice | 6 mice/group | Growth of HSV-tk injected in KF-rb tumors was more suppressed compared with that of KF parental tumors with statistical significance on day 32 (P < 0.05). |
| Therapeutic EphA2 gene targeting in vivo using neutral liposomal small interfering RNA delivery | Landen (2005) | Cancer Research | DOPC (1,2- dioleoyl-sn-glycero-3-phosphatidylcholine) liposome | EphA-targeting siRNA with DOPC liposomes and Paclitaxel | Female athymic nude mice (NCr-nu) bearing HeyA8 and SKOV3ip1 ovarian tumor cells | 10 mice/group | EphA2-targeting siRNA-DOPC (150 Mg/kg twice weekly) reduced tumor growth when compared with a control siRNA (SKOV3ip1: 0.35 vs 0.70 g; P = 0.020; HeyA8: 0.98 vs 1.51 g; P = 0.16); EphA2-targeting siRNA-DOPC + paclitaxel reduced tumor growth compared to a control siRNA + paclitaxel (SKOV3ip1: 0.04 vs 0.22 g; P < 0.001; HeyA8: 0.21 vs 0.84 g; P = 0.0027) |
| Focal adhesion kinasetargeting using in vivo short interfering RNA delivery in neutral liposomes for ovarian carcinoma therapy | Halder (2006) | Clinical Cancer Research | DOPC (1,2- dioleoyl-sn-glycero-3-phosphatidylcholine) liposome | FAK siRNA with DOPC liposomes + docetaxel | Female athymic nude mice (NCr-nu) bearing SKOV3ip1 and HeyA8 ovarian tumor cells | 10 mice/group | FAK siRNA-DOPC in combination with docetaxel reduced mean tumor weight by 94-98% in both SKOVip1 and HeyA8 mice compared to the control group (ANOVA P<0.001) |
| Intraperitoneal delivery of liposomal siRNA for therapy of advanced ovarian cancer | Landen (2006) | Cancer Biology and Therapy | DOPC (1,2- dioleoyl-sn-glycero-3-phosphatidylcholine) liposome | IP EphA2‑targeting siRNA with DOPC liposomes and Paclitaxel | Female athymic nude mice (NCr-nu) bearing HeyA8 and SKOV3ip1 ovarian tumor cells | 10 mice/group | EphA2‑targeting siRNA‑DOPC and paxlitaxel reduced tumor growth by 48–81% compared to paclitaxel/control siRNA‑DOPC IP (HeyA8: 0.34 g v 0.66 g; SKOV3ip1: 0.04 v 0.21, p < 0.01) |
| In vitro and in vivo evaluation of novel cationic liposomes utilized for cancer gene therapy | Serikawa (2006) | Journal of Controlled Release | Cationic liposome | HSV-tk gene and GCV with cationic liposomes | HRA or mEIIL cells xenograft to five-week-old female CD-1 nu/nu athymic nude mice | HRA ascitic mice = 12;mEIIL ascitic mice = 8 | In both HRA and mEIIL mice, mean survival of the HSV-tk treatment group was significantly longer than that of the lacZ treatment group (p<0.05) |
| Efficient inhibition of intraperitoneal human ovarian cancer growth and prolonged survival by gene transfer of vesicular stomatitis virus matrix protein in nude mice | Lin (2007) | Gynecologic Oncology | Cationic liposome | VSVMP-cDNA plasmid with cationic liposomes | Female athymic nude mice (BALB/C) bearing SKOV3 ovarian tumor cells | 3 mice/group | VSVMP-p reduced intraperitoneal tumor weight by about ∼90% compared with control agents (p < 0.01) and significantly prolonged the survival of tumor-bearing mice (p < 0.05) |
| Effect of interleukin-8 gene silencing with liposome-encapsulated small interfering RNA on ovarian cancer cell growth | Merritt (2008) | Journal of the National Cancer Institute | DOPC (1,2- dioleoyl-sn-glycero-3-phosphatidylcholine) liposome | IL-8 siRNA with DOPC liposomes and docetaxel | Female athymic nude mice bearing HeyA8, SKOV3ip1, and SKOV3ip2.TR ovarian tumor cells | 10 mice/group | IL-8 siRNA-DOPC reduced the mean tumor weight by 32% (95% confidence interval [CI] = 14% to 50%; P = .03) and 52% (95% CI = 27% to 78%; P = .03) in the HeyA8 and SKOV3ip1 mice respectively. Treatment with IL-8 siRNA-DOPC plus docetaxel reduced tumor growth in HeyA8 and SKOV3ip1 mice compared to control (77% to 98% reduction in tumor growth; P < .01 for all). |
| Nonviral Nanoscale-Based Delivery of Antisense Oligonucleotides Targeted to Hypoxia-Inducible Factor 1α Enhances the Efficacy of Chemotherapy in Drug-Resistant Tumor | Wang (2008) | Clinical Cancer Research | PEGlyated liposome | HIF1A mRNA antisense oligonucelotides with PEGylated liposome | Female athymic nu/nu mice bearing A2780/AD multidrug-resistant ovarian tumor cells | Not reported | ASO targeted to HIF1A mRNA enhanced the induction of programmed cell death by doxorubicin. The apoptosis induction by the liposomal doxorubicin-ASO combination was >30-fold higher when compared with control, and 4-fold and >1.5-fold higher when compared with the treatment by free doxorubicin and liposomal doxorubicin, respectively. |
| Efficient Inhibition of Ovarian Cancer Growth and Prolonged Survival by Transfection with a Novel Pro-Apoptotic Gene, hPNAS-4, in a Mouse Model | Yang (2008) | Oncology | Cationic liposome | hPNAS-4- p with cationic liposomes | Female BALB/c nude mice (6–8 weeks old) bearing SKOV3 ovarian cell tumors | 5 mice/group | Treatment with hPNAS-4-p significantly delayed growth compared with controls (p < 0.05). After 8 treatment courses, tumor volumes in the control groups were as follows (means + SE): 259.69 + 19.24 mm (PBS), 144.40 + 16.32 mm 3 (LIPO) and 183.46 + 14.63 mm 3 (e-p), being significantly larger than the volume in the hPNAS-4-ptreated group (40.18 + 5.85 mm). |
| Efficient inhibition of cisplatin-resistant human ovarian cancer growth and prolonged survival by gene transferred vesicular stomatitis virus matrix protein in nude mice | Zhong (2008) | Annals of Oncology | Cationic liposome | VSVMP-cDNA with cationic liposomes | Female athymic BALB/c nude mice (6–8 weeks old) bearing A2780s and A2780cp ovarian cell tumors | 5 mice/group | In A2780s tumor model, the apoptotic cells accounted for 44.6% in VSVMP-treated group versus 13.7% in e-p-treated group and 6.2% in NS-treated group. In A2780cp tumor model, the apoptotic cells accounted for 46.1% in VSVMP-treated group versus 11.6% in e-p-treated group and 5.1% in NS-treated group |
| Liposomal siRNA for ovarian cancer | Mangala (2009) | Therapeutic Applications of RNAi | DOPC (1,2- dioleoyl-sn-glycero-3-phosphatidylcholine) liposome | EphA2 siRNA with DOPC liposomes and paclitaxel | Female athymic nude mice bearing HeyA8 ovarian tumor cells | 10 mice/group | Paclitaxel + EphA2 siRNA had the lowest mean tumour weight (0.21g) compared to empty liposomes (2.40g) (p<0.05) |
| Dual targeting of EphA2 and FAK in ovarian carcinoma | Shahzad (2009) | Cancer Biology and Therapy | DOPC (1,2- dioleoyl-sn-glycero-3-phosphatidylcholine) liposome | EphA2 + FAK siRNA with DOPC nanoliposomes | Female athymic nude mice bearing HEYA8 and SKOV3ip1 ovarian tumour cells | 10 mice/group | In SKOV3ip1 and HeyA8 mice treated with EphA-2 plus FAK siRNA-DOPC showed significant reduction in tumor growth compared to control siRNA-DOPC (SKOV2ip1: 76%, p<0.007, HeyA8: 90%, p<0.003) |
| A novel hTERT promoter-driven E1A therapeutic for ovarian cancer | Xie (2009) | Molecular Cancer Therapeutics | Cationic liposome | hTERT-VISA-E1A with cationic liposomes | Luciferase-expressing SKOV3ip1 cells xenograft to athymic female BALB/c nu/nu mice | 12 mice/group | SKOV3ip1 mice treated with T-VISA-E1A showed low luciferase signals and inhibition in tumor growth compared to the control vector (p<0.05). |
| VEGF-Targeted Short Hairpin RNA Inhibits Intraperitoneal Ovarian Cancer Growth in Nude Mice | Bai (2010) | Oncology | Cationic liposome | pGenesil-2-VEGF-shRNA with cationic liposomes | Female athymic nude mice (BALB/c) bearing SKOV3 ovarian tumor cells | 5 mice/group | Mean tumor weight was 0.42 8 0.16, 0.80 8 0.20 and 1.06 8 0.26 g in the VEGF-shRNA-Lipo, HK-shRNALipo and untreated groups, respectively (p < 0.05) |
| Targeting aldehyde dehydrogenase cancer stem cells in ovarian cancer | Landen (2010) | Molecular Cancer Therapeutics | DOPC (1,2- dioleoyl-sn-glycero-3-phosphatidylcholine) liposome | ALDH1A1 siRNA with DOPC liposomes and Docetaxel | Female athymic nude mice bearing SKOV3TRip2 or A2780cp20 ovarian tumor cells | 10 mice/group | ALDH1A1-silencing siRNA significantly reduced growth by 93.6% compared with control siRNA (P < 0.001); |
| Stress effects on FosB- and interleukin-8 (IL8)-driven ovarian cancer growth and metastasis | Shahzad (2010) | Journal of Biological Chemistry | DOPC (1,2- dioleoyl-sn-glycero-3-phosphatidylcholine) liposome | IL-8 siRNA or FosB siRNA with DOPC nanoliposomes | Non stress or stress (norepinephrine and epinephrine) induced female athymic nude mice bearing HEYA8 and SKOV3ip1 ovarian tumour cells | 10 mice/group | In stressed setting, both IL8 and FosB siRNA-DOPC completely blocked the stress-stimulated tumor growth in HeyA8 and SKOV3ip1 mice. In non-stress setting, SKOV3ip1 mice treated with IL8 siRNA-DOPC showed 41% reduced tumor weight (p<0.006), and SKOV3ip1 mice treated with FosB siRNA-DOPC showed 51% reduction in tumor nodules (p<0.01) compared to control siRNA-DOPC. |
| Sustained small interfering RNA delivery by mesoporous silicon particles | Tanaka (2010) | Cancer Research | DOPC (1,2- dioleoyl-sn-glycero-3-phosphatidylcholine) liposome | EphA2 siRNA with cationic liposomes and mesoporus silicon particle | Female athymic nude mice (NCr-nu) (4-5 weeks old) bearing SKOV3ip1 or HeyA8 ovarian tumor cells | 10 mice/group | Treatment with S1MP-EphA2- siRNA-DOPC in mice with SKOV3ip1 tumor significantly reduced tumor weight by 54.2% and 65.3% compared with nonsilencing control siRNA-DOPC and S1MP- nonsilencing control-siRNA-DOPC, respectively (P < 0.05; ANOVA F = 4.92). |
| Cloning of WWOX Gene and Its Growth-inhibiting Effects on Ovarian Cancer Cells | Xiong (2010) | Journal of Huazhong University of Science and Technology - Medical Science | Cationic liposome | pCMV-WWOX with cationic liposomes | A2780 cell line | Not reported | The growth of A2780 cells in the control group was active and the empty vector transfer did not affect cell growth (P>0.05). However, 24, 48, 72 and 96 h after transfection with pCMV-WWOX, the growth of A2780 cells was reduced significantly, with cellular proliferation inhibition rates being 16.41%–38.49% (P<0.01) |
| Therapeutic targeting of PELP1 prevents ovarian cancer growth and metastasis | Chakravarty (2011) | Clinical Cancer Research | DOPC (1,2- dioleoyl-sn-glycero-3-phosphatidylcholine) liposome | PELP1-siRNA with DOPC liposomes | Female athymic nude mice bearing SKOV3ip1 ovarian tumor cells | 8 mice/group | Reduction in metastatic tumor nodules (54%, P < 0.001), reduction in tumor growth (51%, P < 0.001) |
| Silencing of p130Cas in ovarian carcinoma: A novel mechanism for tumor cell death | Nick (2011) | Journal of the National Cancer Institute | DOPC (1,2- dioleoyl-sn-glycero-3-phosphatidylcholine) liposome | p130cas siRNA with DOPC liposomes and docetaxel | Female athymic nude mice bearing HeyA8, SKOV3ip1, and SKOV3ip2.TR ovarian tumor cells | 10 mice/group | Treatment with p130cas siRNA-DOPC in combination with docetaxel chemotherapy resulted in reduced tumor growth compared to siRNA therapy (92%–95% reduction; P < .001). p130cas siRNA-DOPC reduced SKOV3ip1 cell proliferation (31% reduction; P < .001) and increased apoptosis (143% increase, P < .001) compared to control, in vivo. |
| Enhancing chemotherapy response with Bmi-1 silencing in ovarian cancer | Wang (2011) | PLoS ONE | DOPC (1,2- dioleoyl-sn-glycero-3-phosphatidylcholine) liposome | Bmi-1 siRNA with DOPC nanoliposomes and cisplatin | female athymic nude mice (NCr-nu) bearing CP-20 ovarian cell tumors | 10 mice/group | Treatment with Bmi-1 siRNA alone resulted in significant (∼60%) reduction in tumor weight compared to the control siRNA group. |
| Efficient inhibition of intraperitoneal ovarian cancer growth in nude mice by liposomal delivery of short hairpin RNA against STAT3 | Jiang (2013) | Journal of Obstetrics and Gynaecology Research | Cationic liposome | STAT3 shRNA with cationic liposome | Female athymic nude mice (BALB/C) bearing A2780CP ovarian tumor cells | 5 mice/group | In vivo, the tumor weight was reduced to 13.46% of 5% glucose by shSTAT3/lipoplexes (P < 0.01) |
| Mesoporous silicon particles for sustained gene silencing | Hasan (2013) | Ovarian Cancer | DOPC (1,2- dioleoyl-sn-glycero-3-phosphatidylcholine) liposome | EphA2-siRNA-with DOPC liposomes and nanoporous silicon particles | Female athymic nude mice (NCr-nu; 4–5 weeks old) bearing SKOV3 and HeyA8 ovarian tumor cells | 10 mice/group | Mean tumour weight reduced in both SKOV3 and HeyA8 ovarian cancer mouse models (p < 0.05) |
| Folate-linked lipoplexes for short hairpin RNA targeting claudin-3 delivery in ovarian cancer xenografts | He (2013) | Journal of Controlled Release | FRα-targeted lipoplex | CLDN3 shRNA with FRα-targeted lipoplexes | Female athymic nude mice (BALB/3) bearing SKOV3 ovarian tumor cells | 8 mice/group | Mice treated with F-P-LP/CLDN3 reduced tumor weight (90%, p < 0.001) and tumor nodule number (p < 0.001) |
| microRNA‑106a modulates cisplatin sensitivity by targeting PDCD4 in human ovarian cancer cells | Li (2013) | Oncology Letters | Cationic liposome | miR-106a with cationic liposomes | OVCAR3/CIS OCC | Not reported | miR-106a treatment increased miR-106a in the OVCAR3 cells by 7.8-fold and was significantly associated with OCC survival (p<0.05). miR-106a inhibition significantly reduced miR-106a expression and lowered survival rate in the OVCAR3/CIS cell lines (p<0.05). |
| Lipoplex mediated silencing of membrane regulators (CD46, CD55 and CD59) enhances complement-dependent anti-tumor activity of trastuzumab and pertuzumab | Mamidi (2013) | Molecular Oncology | Cationic liposome | 2′-O-methyl sugar-modified anti-CD46, CD55 and CD59 (mCRPs) siRNAs with cationic lipoplexes and trastuzumab and pertuzumab | Female athymic nude mice bearing SKOV3 ovarian tumor cells | 50 μl/well, 2x105 cells/mL | Inhibition of CD59 expression sensitized tumor cells to complement attack (by 27 ± 12% cell lysis in SKOV3 cells, p<0.05), induced an overall cell lysis of 74 ± 13% in SKOV3 cells (p<0.001); Complement-mediated cell death was increased in sensitized cells (p < 0.001 for SKOV3 cells). |
| Therapeutic synergy between microRNA and siRNA in ovarian cancer treatment | Nishimura (2013) | Cancer Discovery | DOPC (1,2- dioleoyl-sn-glycero-3-phosphatidylcholine) liposome | miR-520d-3p with DOPC nanoliposomes | Female athymic nude mice (NCr-nu) bearing HeyA8 and SKOV3ip1 ovarian tumor cells | 10 mice/group | mice bearing miR-520d-3p-overexpressing tumors had a significant reduction in aggregate intraperitoneal metastatic burden as compared with empty vectors (HeyA8-520d-M10 – P < 0.001, and SKOV3ip1– P < 0.05); miR-520d-3p/si-EphA2-1 combination showed a significant reduction in tumor weight compared with miR-520d-3p-DOPC in HeyA8 mice (P ≤ 0.001) |
| Targeted endostatin-cytosine deaminase fusion gene therapy plus 5-fluorocytosine suppresses ovarian tumor growth | Sher (2013) | Oncogene | Cationic liposome | Survivin-VISA-hEndoyCD with cationic liposomes and 5-FC treatment | Female athymic nude mice bearing SKOV3ip1-luc ovarian tumor cells | Ctrl (6); CMV-hEndoyCD (5); SV-hEndoyCD (6) | SV-hENdoyCD reduced tumour weight compared to the control (P < 0.01) |
| EDD enhances cell survival and cisplatin resistance and is a therapeutic target for epithelial ovarian cancer | Bradley (2014) | Carcinogenesis | DOPC (1,2- dioleoyl-sn-glycero-3-phosphatidylcholine) liposome | EDD siRNA with DOPC liposomes and cisplatin | Female athymic nude mice (NCr-nu) bearing ES-2 and A2780ip2 ovarian tumor cells | 10 mice/group | ES-2 reduced tumour burden by 64.4% (P = 0.035) |
| Anticancer effects of chemokine receptor 4(CXCR4) gene silenced by CXCR4-siRNA in nude mice model of ovarian cancer | Liu (2014) | Cell Biochemistry & Biophysics | Cationic liposome | CXCR4 siRNA with cationic liposomes | Female athymic nude mice (BALB/C) bearing SW626 ovarian tumor cells | 8 mice/group | Tumor weight in treatment group were significantly lower than control (P < 0.05); tumor inhibition rate 39.2% compared to 8.9 % in control (P < 0.05) |
| Long Noncoding RNA Ceruloplasmin Promotes Cancer Growth by Altering Glycolysis | Rupaimoole (2015) | Cell Reports | DOPC (1,2- dioleoyl-sn-glycero-3-phosphatidylcholine) liposome | DOPC nanoparticle-incorporated siRNA-mediated silencing of lncRNA alone and with cisplatin | SKOV3 ovarian cancer cells xenograft to 8-12 weeks old female athymic nude mice | 10 mice/group | Cells treated with siNRCP showed significant reduction in cyclin B1 (P<0.05). There was also a significant reduction in cell viability in siNRCP-treated SKOV3 cells compared with sicontrol-treated cells (P< 0.01). |
| Ovarian cancer treatment with a tumor-targeting and gene expression-controllable lipoplex | He (2016) | Scientific Reports | FRα-targeted lipoplex | hTERT promoter-regulated plasmid with FRα-targeted lipoplexes | Female athymic nude mice (NCr-nu) bearing A2780 and SKOV3 ovarian tumor cells | 10 mice/group | F-LP/pMP(2.5) inhibited tumor growth significantly more than other lipoplexes (LP/pVax, P < 0.001; F-LP/pVax, P < 0.01; F-LP/pMP(1), P < 0.01; LP/pMP(2.5), P < 0.05) |
| Anti-EGFR immunonanoparticles containing IL12 and salmosin genes for targeted cancer gene therapy | Kim (2016) | International Journal of Oncology | anti-EGFR immunolipoplex | Plasmid IL12 gene (pIL12) and salmosin gene (pSal) with anti-EGFR immunolipoplexes and Dox | Female athymic nude mice (BALB/C) bearing SKOV3 ovarian tumor cells | 5 mice/group | co-transfection of pIL12 and pSal inihibited tumor growth and pulmonary metastasis (p<0.001); treatment with the anti-EGFR immunolipoplexes containing pIL12/pILSal + doxorubicin resulted in significantly reduced tumor growth (p<0.001) |
| A miR-192-EGR1-HOXB9 regulatory network controls the angiogenic switch in cancer | Wu (2016) | Nat Commun. | DOPC (1,2- dioleoyl-sn-glycero-3-phosphatidylcholine) liposome | miR-192 with DOPC liposomes | Female athymic nude mice bearing SKOV3ip1-NC or SKOV3ip1-miR-192 OCC tumours | 10 mice/group | SKOV3ip1-miR-192 tumours showed a 70% decrease in burden compared to control (p<0.05). Compared with SKOV3ip1-NC tumours, SKOV3ip1-miR-192 tumours showed a significant 55% reduction in MVD (P<0.05). |
| Efficient delivery of Notch1 siRNA to SKOV3 cells by cationic cholesterol derivative-based liposome | Zhao (2016) | International Journal of Nanomedicine | Cationic liposome | Notch1 siRNA/DMAPA-chems nanoparticles (Novel cationic cholesterol derivative-based liposome, DMAPA-chems, for efficient delivery of siRNA into human SKOV3 ovarian cancer cells to inhibit Notch1 gene expression) | SKOV3 cell line | Not reported | Cell growth was significantly inhibited at 24, 48, and 72 hours after treatment with Notch1 siRNA/DMAPA-chems nanoparticles. Treatment with Notch1 siRNA/DMAPA-chems nanoparticles increased the percentage of apoptotic cells (P<0.01). |
| Targeting the centriolar replication factor STIL synergizes with DNA damaging agents for treatment of ovarian cancer | Rabinowicz (2017) | Oncotarget | DOPC (1,2- dioleoyl-sn-glycero-3-phosphatidylcholine) liposome | STIL-specific siRNAs with DOPC nanoliposomes | Female athymic nude mice (8 to 12 weeks old) bearing HeyA8 or IGROV1 ovarian tumor cells | 10 mice/group | siSTIL-DOPC alone resulted in 69% (HeyA8) and 65% (IGROV1) reduction in tumor burden compared to siControl-DOPC treated mice. |
| A polycation coated liposome as efficient siRNA carrier to overcome multidrug resistance | Xia (2017) | Colloids and Surfaces B: Biointerfaces | PDADMAC (polycation) coated liposome | MDR1 siRNA with PDADMAC coated liposomes | OVCAR8 and OVCAR8/ADR cell lines | Not reported | Application of siRMDR1 increased the efficacy of tumor inhibition. siRNA treated at 120 nM, the IC50 of DOX after AL-PDAD-MDR treatment was 1.5 μg/ml, 2-fold lower than that of AL- PDAD-NC and 15 times lower than that of free DOX. The MDR1 siRNA treatment caused stronger tumor inhibition, confirming the effect of gene silencing. |
| Small interfering RNA targeting of the survivin gene inhibits human tumor cell growth in vitro | Zhang (2017) | Experimental and Therapeutic Medicine | Cationic liposome | survivin siRNA with cationic nanoliposome | SK-OV-3 cell line | Not reported | significant inhibition of cell proliferation was detected in all cell lines relative to the si-NC group (P<0.05); (For SKOV3 cell, inhibition rate at 96h was 7.0±0.9 for siRNA group and 0.4±1.8 for si-NC group). |
| In Vivo Ovarian Cancer Gene Therapy Using CRISPR-Cas9 | He (2018) | Human Gene Therapy | Cationic liposome | F-LP carrying CRISPR plasmid DNA coexpressing Cas9 and sgRNA targeting ovarian cancer-related DNMT1 gene (gDNMT1) | Female athymic female nude mice (BALB/c) bearing SKOV-3 ovarian tumor cells | 8 mice/group | Tumor growth inhibition rate of F-LP/gDNMT1 was 53.6% (p < 0.001) in PTX-sensitive tumors and 45.9% in PTX-resistant tumors (p < 0.05) |
| Anticancer activity of the intraperitoneal-delivered DFP-10825, the cationic liposome-conjugated RNAi molecule targeting thymidylate synthase, on peritoneal disseminated ovarian cancer xenograft model | Iizuka (2018) | Drug Design, Development and Therapy | Cationic liposome | Tymidylate synthase shRNA with cationic liposome and paclitaxel | Male SCID mice bearing SKOV3-luc ovarian tumor cells | 10 mice/group | DFP-10825 (1 mg/kg as TS shRNA) resulted in significant tumor growth inhibition with 56%, 70% and 65% on days 14, 21 and 28, respectively (P<0.05) |
| PEGylated DC-Chol/DOPE cationic liposomes containing KSP siRNA as a systemic siRNA delivery Carrier for ovarian cancer therapy | Lee (2018) | Biochemical and Biophysical Research Communications | PEGylated lipoplex | KSP siRNA with PEGylated lipoplexes | Female athymic nude mice (BALB/C) bearing SKOV3 ovarian tumor cells | 3 mice/group | Decrease in the average tumor volume and tumor weight by ~59.6% and ~53.1% (p < 0.001) ; high level of TUNEL-positive SKOV3 cells in the tumor tissues of LS_siKSPinjected group |
| Activating p53 family member TAp63: A novel therapeutic strategy for targeting p53-altered tumors | Gunaratne (2019) | Cancer | DOPC (1,2- dioleoyl-sn-glycero-3-phosphatidylcholine) liposome | miR-130b with DOPC nanoliposomes and a tumor-targeted nanocomplex (scL) | Female athymic nude mice bearing HEYA8 and OVCAR8 ovarian tumor cells | 5 mice/group | miR-130b in DOPC liposomes decreased tumor burden (p < 0.01); 20% of DOPC–miR-130b plus CDDP-treated mice were tumor free |
| Analysis of the inhibitory effects of miR-124 and miR-152 on human epithelial ovarian cancer xenografts in a nude mouse model | Liu (2019) | Oncology Letters | Cationic liposome | miR-124 and miR-152 with cationic liposomes | BALB/c nude mice bearing SKOV3 OCC tumours | 4 mice/group | miRNA-124 treatment significantly decreased tumour volume in mice (7.88±2.84 mm3) vs. control (43.57±20.64 mm3) (p<0.01). miRNA-152 treatment had significantly decreased tumour volume in mice (8.64±3.52 mm3) vs. control (45.74±22.31 mm3) (P<0.01). |
| Surface-engineered polyethyleneimine-modified liposomes as novel carrier of siRNA and chemotherapeutics for combination treatment of drug-resistant cancers | Mendes (2019) | Drug Delivery | bPEI conjugated liposome | MDR1 siRNA with bPEI-modified liposomes and paclitaxel | Female athymic nude mice bearing A2780-ADR ovarian tumor cells | 4 mice/group | Mice treated with 0.5% PEIPOS/PTX/siMDR1 had tumor volumes approximately 40% smaller than controls 18 days after the treatment started (p < 0.0001) |
| PTGER3 induces ovary tumorigenesis and confers resistance to cisplatin therapy through up-regulation Ras-MAPK/Erk-ETS1-ELK1/CFTR1 axis | Rodriguez-Aguayo (2019) | EBioMedicine | DOPC (1,2- dioleoyl-sn-glycero-3-phosphatidylcholine) liposome | MSV-PTGER3-siRNA with DOPC liposomes and cisplatin | Female athymic nude mice (6-8 years old) bearing A2780-CP20 and OVCAR5 ovarian tumor cells. | 4 mice/group | Mice treated with either with DOPC-PTGER3 siRNA, 2’-F-PS2-PTGER3 or MSV-DOPC-PTGER3-siRNA showed significant reduction in cell proliferation compared to control (P*** < 0.0001, P*** < 0.0001, P<0.001). |
| Cytoplasmic expression of EGFR shRNA using a modified T7 autogene-based hybrid mRNA/DNA system induces long-term EGFR silencing and prolongs antitumor effects | Seraj (2019) | Biochemical pharmacology | DOPC lipoplex (cationic) | SKOV3-bearing cell line xenograft mice intravenously received auto_shEGFR@LS, scrambed auto_scEGFR@LS, siEGFR@LS, or PBS twice every two weeks | SKOV3 cells xenograft to female nude mice (BALB/c) | siEGFR group (n=8), auto_shEGFR@LS group (n=8), auto_scEGFR group (n=4) | auto_shEGFR@LS-treated mouse group showed a significant reduction in tumor weights compared to control (67.6% P < 0.001) |
| Anti-EGF antibody cationic polymeric liposomes for delivery of the p53 gene for ovarian carcinoma therapy | Zuo (2019) | International journal of clinical and experimental pathology | EGF conjugated liposome | p53 gene with EGF conjugated liposomes | SKOV3 cell line | Not reported | At increased NP concentrations of 20.0 and 80.0 µg/ml, CPLs/p53 and EGF-CPLs/p53 both significantly enhanced the cytotoxicity (P<0.05) |
| Switching the intracellular pathway and enhancing the therapeutic efficacy of small interfering RNA by auroliposome | Hossen (2020) | Science Advances | Auroliposome | MICU1-siRNA with auroliposomes and cisplatin | ovarian cancer patient-derived tissue xenograft to NOD/SCID mice; 5 PDX tumors that express MICU1 were examined, including PDX-098 | 10 mice/group | Mice treated with MICU1-siRNA AuroLPs and cisplatin showed significant tumor volume and weight inhibition compared to the control (p<0.0001, p<0.001 respectively) |
| Aptamer (AS1411)-Conjugated Liposome for Enhanced Therapeutic Efficacy of miRNA-29b in Ovarian Cancer | Jiang (2020) | Journal of Nanoscience and Nanotechnology | Aptamer-conjugated cationic liposomes | miRNA-29b with AS1411-conjugated cationic liposomes | A2780 OCC | 4×10^4 cells/well | miRNA-29b treatment showed a concentration dependent cytotoxic effect in OCC. |
| Therapeutic efficacy of liposomal Grb2 antisense oligodeoxynucleotide (L-Grb2) in preclinical models of ovarian and uterine cancer | Lara (2020) | Oncotarget | Cationic liposome | Grb2 antisense oligodeoxynucleotide with cationic liposomes | Female athymic nude mice bearing OVACAR5 and SKOV3ip1 cells | 9 mice/group | Treatment with L-Grb2 and paclitaxel led to the greatest decrease in tumor weight (mean ± SEM, 0.17 g ± 0.10 g, p < 0.05) compared with that in control mice (0.99 g ± 0.35 g). Treatment with L-Grb2 alone also showed a decrease in tumor weight (0.29 g ± 0.14 g). |
| KSP siRNA/paclitaxel-loaded PEGylated cationic liposomes for overcoming resistance to KSP inhibitors: Synergistic antitumor effects in drug-resistant ovarian cancer | Lee (2020) | Journal of Controlled Release | PEGylated liposome | KSP siRNA with PEGylated cationic liposomes and paclitaxel | Female athymic nude mice (BALB/C) bearing HEYA8-MDR ovarian tumor cells; Female athymic nude mice (BALB/C) bearing tumor tissues from a 62yo female patient platinum-resistant epithelial ovarian cancer | 5 mice/group; 4 mice/group | siKSP/PTX@lp-injected mice showed decreased tumor weights compared to control (77.5% reduction P < .005), and tumor growth inhibition (81.2% inhibition) compared to control; Tumor growth was significantly inhibited in the siKSP/PTX@lp-injected PDX group when compared with PBS-injected PDX group (33.5% P < .01) |
| MicroRNA-18a-5p Suppresses Tumor Growth via Targeting Matrix Metalloproteinase-3 in Cisplatin-Resistant Ovarian Cancer | Quinones-Diaz (2020) | Frontiers in Oncology | Folate conguated cationic liposome | miRNA-18a-oligonucleotide with folate-conjugated liposomes | NCRNU-F, female athymic nude mice bearing A2780CP20 OCC tumours | 8 mice/group | miRNA-19a-oligonucleotide treatment significantly reduced tumour number and weight compared to control (p<0.05). |
| Downregulation of hTERT contributes to ovarian cancer apoptosis and inhibits proliferation of ovarian cancer cells | Wang (2020) | Translational Cancer Research | Cationic liposome | hTERT interfering gene with cationic liposomes (lipofectamine TM2000) | SKOV3 cell line | Not reported | SKOV3 cells transfected with hTERT shRNA showed significantly slower growth and reduced proliferation compared with SKOV3 cells transfected with empty plasmid on the second and third days after transfection (P<0.05 vs NC group). The total apoptosis rate in the hTERT shRNA group was 18.66±1.33, which was significantly higher than that in the control group (2.92±0.33). (P<0.05 vs. NC group) |
| Liposomal Delivery of MicroRNA-7 Targeting EGFR to Inhibit the Growth, Invasion, and Migration of Ovarian Cancer | Cui (2021) | ACS Omega | Cationic liposome | miR-7 with cationic liposomes | SKOV-3 cell line | 5 × 10^5 cells/well | miR-7 in cationic liposomes suppressed the proliferation of SKOV3 cells comapred to control (P<0.001) |
| Targeting eukaryotic elongation factor-2 kinase suppresses the growth and peritoneal metastasis of ovarian cancer | Erdogan (2021) | Cellular Signalling | DOPC (1,2- dioleoyl-sn-glycero-3-phosphatidylcholine) liposome | EF2K-siRNA with DOPC liposomes | Female athymic nude NcR mice bearing SKOV3ip1 ovarian tumor cells | 5 mice/group | Mice treated with liposomal EF2K siRNA had significantly reduced umtour size and fewer tumor nodules compared to control siRNA (P<0.05) |
| LINC00184 Promotes Ovarian Cancer Cells Proliferation and Cisplatin Resistance by Elevating CNTN1 Expression via Sponging miR-1305 | Han (2021) | OncoTargets and Therapy | Cationic liposome | LINC00184 shRNA with cationic liposomes | A2780-DDP cells xenograft to nude mice | 6 mice/group | Mice treated with shLINC00184 reduced tumour weight and volume (P<0.01) |
| Expression of Cyclin D1 gene in ovarian cancer and effect of silencing its expression on ovarian cancer cells based on the Oncomine database | Quan (2021) | Bioengineered | Cationic liposome | CCND1-siRNA with cationic liposomes | SKOV-3 cell line | 1x10^5 cells/mL | CCND1-siRNA showed a signficantly higher apoptosis rate of SKOV3 ovarian cancer cells compared to control (P<0.01) |
| Long non-coding RNA miR155HG silencing restrains ovarian cancer progression by targeting the microRNA-155-5p/tyrosinase-related protein 1 axis | Wen (2021) | Experimental and Therapeutic Medicine | Cationic liposome | miR155HG siRNA with cationic liposomes | OVCAR3 and SK-OV-3 cell line | 2x10^3/well | OVCAR3 and SKOV-3 cells transfected with miR155HG siRNA showed reduced cell viability (P<0.01) and supressed tumor migration and invasion (P<0.001) compared to control |
| Investigating function of long noncoding RNA of HOTAIRM1 in progression of SKOV3 ovarian cancer cells | Ye (2021) | Drug Development Research | Cationic liposome | HOTAIRM1 siRNA with cationic liposomes | SKOV-3 cell line | 2 x10^5 cells/well | HOTAIRM1 siRNA treatment showed significantly higher number of apoptotic cancer cells compared to control siRNA (P<0.05); treatment also significantly decreased in tumor cell growth and proliferation (P<0.05) |
| Exosome-liposome hybrid nanoparticle codelivery of TP and miR497 conspicuously overcomes chemoresistant ovarian cancer | Li (2022) | Journal of Nanobiotechnology | integrin alpha-v and beta-3 targetted liposome (modified with cRGD) | miR497/triptolide with a combination of SKOV3-CDDP exosomes and cRGD modified liposomes (HENPs) | BALB/c-nu mice bearing subcutaneous SKOV3-CDDP tumors | 3 mice/group | Significant suppression of tumor growth (87%) was detected in the miR497/TP-HENPs group compared to the naked miR497 and TP (P<0.001). Tumor volume was also lowest in the miR497/TP-HENPs group with an average volume of 107 ± 27 mm3. |
| Upregulation of the Long Noncoding RNA CASC10 Promotes Cisplatin Resistance in High-Grade Serous Ovarian Cancer | Rivera (2022) | International Journal of Molecular Sciences | DOPC-based nanoliposomes | CASC10 siRNA and cisplatin with DOPC liposomes | Female aythmic mice (NCr-nu) bearing OVCAR3CIS ovarian cell tumors | 10 mice/group | Tumor weight and number of tumor nodules were significantly lower in CASC10-siRNA group compared to the NC-siRNA or cisplatin group (P<0.05). Effects were exacerbated when the CASC10-siRNA treatment was combined with cisplatin (P<0.008). |
| Knockdown of lncRNA ACTA2-AS1 reverses cisplatin resistance of ovarian cancer cells via inhibition of miR-378a-3p-regulated Wnt5a | Lin (2022) | Bioengineered | Cationic liposomes (Lipofectamine 2000) | siACTA2-AS1 with cationic liposomes | A2780/DDP and SKOV3/DDP cells | 5 × 10^3/well | In comparison to the si-NC group, the IC50 value of DDP was lower in the si-ACTA2-AS1 group (P<0.05). In vitro, knocking down ACTA2-AS1 decreases DDP resistance in OC cells. |
| The Therapeutic Effects of DDP/CD44-shRNA Nanoliposomes in AMF on Ovarian Cancer | Guo (2022) | Gynecological Oncology | PEG-modified manganese zinc ferrite nanoparticles nanoliposomes | PEG-MZF-NPs/DDP/CD44-shRNA nanoliposomes | Six-week-old female BALB/C nude mice bearing Ovarian Cancer HO8910 Cells | Not reported | The combination of DDP/CD44-shRNA/MFH group reported significantly better tumor mass and volume inhibition rates of (92.80 ± 1.09)% and (89.02 ± 6.68)%, respectively, in comparison to the individual treatment groups [DDP group: (40.82 ± 5.66)% and (16.10 ± 2.79)%; DDP/CD44-shRNA group: (56.53 ± 2.40)% and (36.21 ± 6.99)%; MFH group: (70.63 ± 5.94)% and (54.95 ± 8.93)%; CD44-shRNA/MFH group: (79.59 ± 1.59)% and (64.67 ± 3.84)%; and DDP/MFH group: (86.49 ± 5.36)% and (80.35 ± 2.73)%, p<0.05] |
| Inositol monophosphatase 2 promotes epithelial ovarian cancer cell proliferation and migration by regulating the AKT/mTOR signaling pathway | Ablimit (2022) | Experimental and Therapeutic Medicine | Cationic liposomes (Lipofectamine 2000) | shIMPA2 with cationic liposomes | Female BALB/c nude mice bearing ES-2 cells | 5 mice/group | shIMPA2 with liposomes significantly reduced tumorigenicity by reducing tumor volume and weight (P<0.001) |
| NANO-SBT-PEDF delivery system: A promising approach against ovarian cancer? | Ribaux (2023) | Heliyon | Cationic liposomes | PEDF pDNA with cationic liposomes | SKOV3 and COV318 cells | 1.5x10^4 cells/well | PEDF pDNA with the liposome 2 (DOTAP/cholesterol 3/1M ratio) significantly reduced relative cell viability compared to coontrol (P<0.0001) in both SKOV3 cells and COV318 cells. Insignificant outcomes were found in liposomal carriers without implementation of cholesterol. |
